# Transcriptome Analysis for Identification of Genes Related to Growth and Development, Digestion and Detoxification, Olfaction in the Litchi Stink Bug *Tessaratoma papillosa*

**DOI:** 10.3389/fphys.2021.774218

**Published:** 2022-01-24

**Authors:** Lin Cheng, Shuncai Han, Jingtao Jiang, Haichao Li, Lingfei Peng

**Affiliations:** ^1^Biological Control Research Institute, Fujian Agriculture and Forestry University, China Fruit Fly Research and Control Center of FAO/IAEA, Key Laboratory of Biopesticide and Chemical Biology, Ministry of Education, State Key Laboratory of Ecological Pest Control for Fujian and Taiwan Crops, Fuzhou, China; ^2^Chinese Academy of Sciences Key Laboratory of Insect Developmental and Evolutionary Biology, CAS Center for Excellence in Molecular Plant Sciences, Shanghai Institute of Plant Physiology and Ecology, Chinese Academy of Sciences, Shanghai, China

**Keywords:** *Tessaratoma papillosa* (Drury), transcriptome, olfactory genes, growth and development, digestion and detoxification

## Abstract

*Tessaratoma papillosa* is a major pest of *Litchi chinensis* and *Dimocarpus longan*. Adult and nymph secretions are not only harmful to plants but also to humans. At present, there are not a lot of research on *T. papillosa*, especially omics research. We used high-throughput sequencing technology to sequence the *T. papillosa* transcriptome and obtained 67,597 unigenes homologous to *Halyomorpha halys* (88.03%). Subsequently, RNA-SEQ and comparative analyses were performed on the 14 different developmental stages and tissues. A total of 462 unigenes related to growth and development, 1,851 unigenes related to digestion and detoxification, and 70 unigenes related to olfaction were obtained. Moreover, expression analysis showed that the *T. papillosa* major life activities genes are uniformly expressed across all developmental states. However, the adult midgut gene expression patterns were utterly different from that of the nymphs. Similarly, female fat body genes exhibited distinct expression patterns compared to that of males and nymphs. Thus, different developmental stages and physiological functions affect gene expression patterns. We also found that most of the differential genes were associated with cellular maintenance. This study will help understand the growth and development of litchi stink bugs, their choice of host plants, food digestion and detoxification, and their reproductive behavior. In addition, this result can provide reference information for some target genes in the process of control of *T. papillosa*.

## Introduction

The selection of host plants by herbivorous insects relies on sensory signals, including vision, smell, taste, and touch. These signals enable the insects to distinguish the plants’ physical properties and accurately identify the secondary plant metabolites and subsequent detoxification (Mollo et al., 2017). Furthermore, the insects locate their host plants through chemotaxis. The plants release signal stimuli which are then detected by the insects (Finch and Collier, 2000; Yang et al., 2001). For example, the insect odor binding protein senses and transmits odor substances and regulates oviposition. The larvae of *Helicoverpa armigera* and *Helicoverpa assulta* have the habit of feeding on each other. Meanwhile, male moths transmit the OBP10 carrying 1-dodecene to female moths through mating. After mating, the 1-dodecene is then located on the egg surfaces laid on host plants. Consequently, moths detect this compound through the OBP10 in their antennae and avoid the plants containing the eggs; this mechanism also helps prevent cannibalism (Sun et al., 2012) at the same time, to counter the effects of the plant metabolites, insects have evolved various physiological and biochemical adaptation mechanisms. The emergence of a series of detoxification enzymes is one of the defense mechanisms developed by insects against plant metabolites (Oakeshott et al., 2003; Clarkson et al., 2018). A few insects store toxic substances from plants to avoid poisoning and enhance their food utilization efficiency and selection of host plants. Additionally, the intrinsic factors regulating this mechanism mainly depend on the abundance and expression patterns of the insects’ digestive and detoxification enzyme genes (Yang et al., 2001). These genes play a vital role in the growth and development of insects, and they are also important target genes in pest control.

Obtaining the omics data of insects is useful for understanding the growth and development of insects and screening targeted control targets. Rapid development of high-throughput sequencing technologies has greatly improved research on non-model insects, making the acquisition of their genetic, physiological, and ecological information easier. For instance, Wu et al. (2017) used transcriptome sequencing technology to analyze the *T. papillosa* olfaction gene families. Moreover, targeted acquisition of pest control strategies based on molecular targeting technology offers a viable alternative approach to mitigating pests (Delye et al., 2020; Schmidt et al., 2021).

**FIGURE 1:**
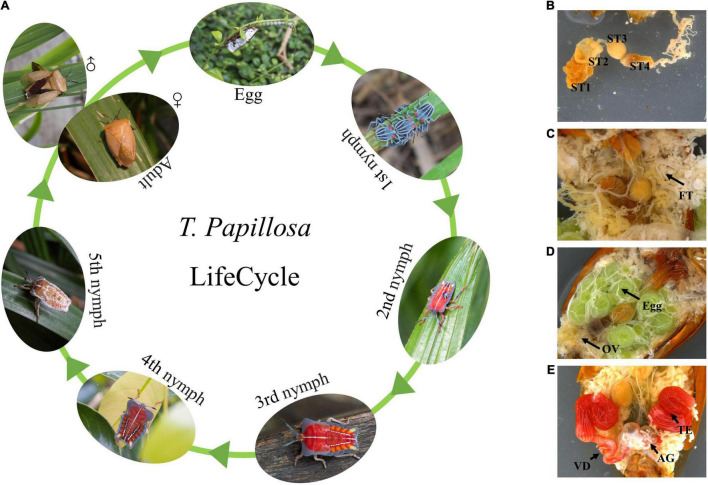
Morphology of *T. papillosa* at different developmental stages and organization Chart. **(A)** *T. papillosa* Life cycle. **(B)** reproductive system of female adult. **(C)** Digestive tract. **(D)** Internal organs. **(E)** reproductive system of male adult.

*Tessaratoma papillosa* is an important pest of longan and lychee, mainly distributed in Southeast Asia (Thailand and Vietnam), India and southern China (Guangdong, Fujian, Guangxi, and Taiwan) (Li et al., 2014; Tran et al., 2019; Wu et al., 2020). *T. papillosa* is also an important pest for greening trees. At the same time, when exposed to external stimuli, it releases toxic liquids, irritating human skin and eyes, and causing diseases (Zhang et al., 2009). It is also the vector insect of soot disease and *Longan witches broom-associated virus* (LWBD) (Chen et al., 2001; Meng et al., 2017). *T. papillosa* has a relatively long life cycle, and the entire life cycle can be harmful, which brings great difficulties to its prevention and control (Figure 1A). At present, chemical pesticides are not effective in the prevention and control of *T. papillosa*, and there are fewer natural enemies of *T. papillosa*, which makes prevention and control difficult. Recent advancements in RNAi technology have, however, made the control of pathogens such as *T. papillosa* possible (Jain et al., 2021).

In this study, the deep transcriptome sequencing technology is used to explore the growth and development-related genes in different developmental stages and tissues of *T. papillosa*. The findings might be helpful in understanding *T. papillosa* and other stink bugs survival traits, such as spawning and reproductive cycles, choice of host plants, and food digestion mechanisms.

## Materials and Methods

### Insects and Samples

Approximately 230 *T. papillosa* bugs were collected on the wild Lychee trees from four Lychee orchards in Fuzhou, Fujian Province, China. All the bugs were bring back to the lab alive, then the wings, legs, scutellum, and abdominal tergum were cut off with scissors, the abdominal tergum were removed carefully, and the viscera were exposed. The body was fixed with insect pin on a wax plate, and dissected under a stereo-microscope. We collected 14 samples including female and male adults, nymph of different instars, antennae of adults and nymphs, ovaries and testis, fat body (female and male adult, nymphs), midgut (female and male adult, nymphs), and hemolymph. Each samples were mixed with five bugs tissues or organs. After mixing, the samples were immediately frozen in liquid nitrogen for 10 min and then stored at −80°C for RNA extraction. For each samples, experiments were performed in triplicates (Supplementary Table 1).

### RNA Extraction, Library Construction, and Sequencing

RNA was extracted from the 14 samples using the TRIzol extraction method and then purified using the RNeasy^®^ MinElute^®^ cleanup kit (Qiagen^®^). RiboZeroTM Magnetic (Plant leaf) kit (Epicentre^®^) was used to degrade rRNA from 4 μg of each sample. The removal of rRNA was then verified using a 2100 BioAnalyzer (Agilent Technologies).

After total RNA extraction, mRNA was enriched using oligo dT magnetic beads, and the fragmentation buffer was used to break the mRNA into shorter fragments. Messenger RNA was utilized as a template for the first cDNA strand synthesis using a six-base random primer. Subsequently, dNTP, RNase H, and DNA polymerase I were used for subsequent cDNA strands synthesis. End-repair was then conducted after purification by the QIAquick PCR kit and elution with EB plus foya buffer. Eventually, gel electrophoresis was done for fragment selection, followed by PCR amplification and the constructed library sequenced using BGI-500 (BGI), three replicates per sample.

### Transcriptome Assembly, Gene Annotation, and Ontology

We used the short reads assembly software SOAPdenovo (Li et al., 2010) to perform a *de novo* transcriptome assembly. SOAPdenovo concatenates reads with a certain length of overlap into longer N-free fragments called Contigs. We then compared paired-end reads between the obtained reads and the contigs to determine the variant contigs that originated from the same transcript and the distance between these contigs. Usually, SOAPdenovo links these contigs together, and the intermediate unknown sequences are denoted by N, so that scaffold. The paired-end reads were then used to generate the scaffold with the least N content and shorter extension at both ends. This scaffold was then referred to as unigene. If multiple samples from the same species are sequenced, the unigene can be further sequenced and de-duplicated by the sequence clustering software to obtain the longest and non-redundant consensus unigene. Finally, the unigene sequence was aligned with sequences from the protein databases, including NT, Swiss-Prot, KEGG, and COG databases (e-value < 0.00001), and the best-aligned protein was determined by the Unigene sequence orientation. In case of contradicting comparison results between different libraries, the unigene sequence direction was obtained according to the priority Scores by the NR, Swiss-Prot, KEGG, and COG databases. We used the software ESTScan (Iseli et al., 1999) to predict the coding region and determine the sequence direction for a unigene sequence that was incomparable with the four databases. Moreover, the unigene whose sequence direction was, determined was read in the 5′ to the 3′ direction, whereas the unigene with undetermined sequence direction was read based on the denotations generated by the assembly software.

### Sequence Analysis

Differential expression analysis was performed using DESeq R package (1.10.1), which is based on a negative binomial distribution model. DESeq is a statistical program that determines the differential expression in digital gene expression data using a model-based negative binomial distribution (Sun M. et al., 2020), Meanwhile, the *P* values were adjusted using Benjamini and Hochberg’s approach to account for the false discovery rate (FDR). Genes with a twofold difference (the absolute value of log2 ratio ≥ 1) between two stages and an FDR < 0.01 were categorized as differentially expressed genes (DEGs).

### qRT-PCR Assays

Total RNA was isolated from the collected samples using the TRIzol reagent (Invitrogen). The RNA served as the template for synthesizing cDNA using the First Strand cDNA Synthesis Kit (Toyobo). The qRT-PCR analysis was performed using the SYBR Green Real-time PCR Master Mix Kit (Toyobo). Details regarding all primers are listed in Supplementary Table 7. Melting-curve analyses were performed for all of the primers. To normalize Ct values obtained for each gene, actin-2 expression levels were used. RT-qPCR was carried out using Mastercycler^®^ ep realplex (Eppendorf). All qRT-PCR assays were repeated three times. qRT-PCR reactions and data were analyzed according to the methods of Livak and Schmittgen (2001) and Bustin et al. (2009). The data were analyzed using a one-way analysis of variance (ANOVA) to look for treatment effects compared with the untreated control.

## Results

### *T. papillosa* Midgut Morphology

Mitgut of *T. papillosa* contains four parts (four stomachs), the first part was the largest one, brown in color and pouched in shape; the second part was short and tubular; the third part was spherical; the fourth part was the longest one, which is up to two times of the body length, and can be separated into two parts, one pouch and followed by a long thin tube (Figure 1B). The fat body was milky white, resembled grapes in shape (Figure 1C). Female adults had a pair of ovaries, each ovary composed of seven ovarioles (Figure 1D) and male adults possessed a pair of red kidney-shaped testicles with deferens ducts (Figure 1E).

### *T. papillosa* Transcriptome Analysis

A total of 7.29 Gb data was obtained. Additionally, the normalized data were regarded as clean if the reads had Q20 of 96.8%, Q30 of 88.11%, and the ratio of 89.19%. Clean reads were assembled, and 67,597 unigenes were obtained after de-redundancy. The total length, average length, N50, and GC content were 74, 010, 888 bp, 1,094 bp, 2,016 bp, and 35.13%, respectively. Moreover, unigene length ranged between 200 and 16,160 bp, with an average of 200–300 bp (16, 754). There were 5,224 unigenes with 3,000 or more base pairs (Supplementary Figure 1A).

**FIGURE 2:**
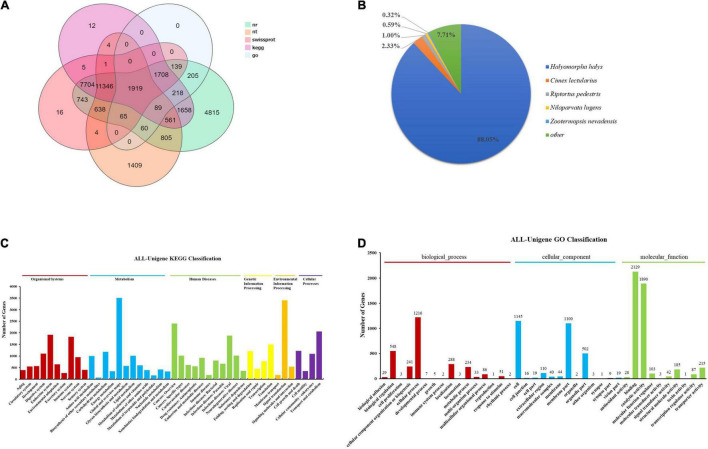
Overall analysis of transcriptome data of *T. papillosa*. **(A)** Five gene function annotation database annotation results Venn diagram. **(B)** NR annotated species distribution. **(C)** The abscissa indicates the number of corresponding transcripts, and the ordinate indicates the functional classification of KEGG. **(D)** The abscissa indicates the number of corresponding transcripts, and the ordinate indicates the GO function classification.

Eventually, 32,673 unigenes accounting for 48.33%, 16,901 unigenes accounting for 25.00%, 24,288 unigenes accounting for 35.93%, 23,401 unigenes accounting for 34.62%, 25,225 unigenes accounting for 37.32%, 4,403 unigenes accounting for 6.51%, and 24,097 unigenes accounting for 35.65% were deposited to the NR, NT, SwissProt, KOG, KEGG, GO, and Pfam databases, respectively (Figure 2A). Transdecoder software was then used to detect 27, 031 CDS (Supplementary Figure 1B). Annotated gene species similarity results showed that *T. papillosa* and *Halyomorpha halys* had more similar genes (88.05%), followed by *Cimex lectularius* (2.33%), *Riptortus pedestris* (1.00%), *Nilaparvata lugens* (0.59%), and then *Zootermopsis nevadensis* (0.32%) (Figure 2B).

KEGG enrichment analysis of all unigenes and obtained 45 pathways, with the ubiquitin-mediated proteolysis being abundantly represented (Figure 2C). The five pathways with the highest number of unigene enrichment hits were (1) Global and overview maps (3,509), (2) Signal transduction (3,404), (3) Cancers: Overview (2,399), (4) Transport and catabolism (2,055), and (5) Endocrine system (1,915). Additionally, 10,442 unigenes were assigned to the three GO categories: biological processes, cellular components, and molecular functions (Figure 2D). In the biological processes category, level 2 terms cellular processes (1,216) and biological regulation (548) were the most frequently represented, whereas, in the cellular component category, level 2 terms cellular processes (1,145) and membrane (1,100) were the predominant categories. Meanwhile, binding (2,129) and catalytic activities (1,890) were the most abundant processes in the molecular function category. During the comparison and analysis of all unigenes in the Animal TFDB 2.0 database, 4,101 transcription factors were annotated. Six transcription factors families [zf-C2H2(862), RHD(275), TF_bZIP(252), Homeobox(222), HGM(209), and MYB(186)] with the most significant number of unigenes accounted for 48.91% of all transcription factors (Supplementary Figure 2).

### Analysis of Differentially Expressed Genes in Different Developmental Stages and Tissues of *T. papillosa*

RNA-SEQ sequencing obtained reads with than 21M (number of bases > 1 Gb) from each set. Both Q20 and Q30 were more than 90% (Supplementary Table 2). The proportion of sequences mapped to the transcriptome data was between 88.92 and 95.57%, and the unique, clean reads were 36.20–56.91% (Supplementary Table 3). Meanwhile, pairwise comparison results showed that the correlation coefficient between the samples was between 0.1907 and 0.9708 (Supplementary Figure 3A). Moreover, PCA analysis showed that the nymph, female and male midguts formed a separate cluster from the female ovary and the fat body cluster. The nymph, male antennae, and testis, however, clustered separately. The results were consistent with the physiological functions of the genes expressed in different body parts, depicting significant differences in the functional structure between different developmental stages tissues (ANOSIM, *P* < 0.001). In addition, t gene expression had a normal distribution, indicating the occurrence of a normal gene expression distribution state (Supplementary Figure 3C). Considering the dynamic changes associated with gene expression, the co-expression between genes was calculated, and the gene expression regulation model containing essential genes at different developmental stages or under different conditions was searched. Weighted Correlation Network Analysis (WGCNA) results showed that the correlation between genes within the same module was very high but much lower between genes in different modules (Supplementary Figure 3D). Different *T. papillosa* samples exhibited a direct correlation between the gene expression patterns and the physiological function of tissues during *T. papillosa* development. According to PCA analysis, the nymph, female and male midguts formed a separate cluster from the female ovaries and fat body. Moreover, the correlation coefficient analysis showed that samples with large correlation variations have different physiological functions. Expression analysis also showed that most genes were expressed across the different developmental states.

**FIGURE 3:**
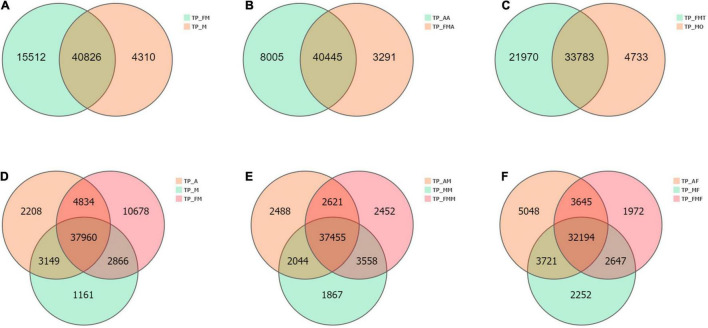
Venn diagram of the number of differential genes between different tissue samples. **(A)** Comparative analysis of female (TP_FM) and male adult (TP_FM). **(B)** Comparative analysis of nymph antennae (TP_AA) and adult antennae (TP_FMA). **(C)** Comparative analysis of ovaries (TP_MO) and testis (TP_FMT). **(D)** Comparative analysis of nymphs (TP_A), female adult (TP_FM) and male adult (TP_M). **(E)** Comparative analysis of nymphs midgut (TP_AM), female adult midgut (TP_FMM) and male adult midgut (TP_MM). **(F)** Comparative analysis of nymphs fat body (TP_AF), female adult fat body (TP_FMF) and male adult fat body (TP_MF).

**FIGURE 4:**
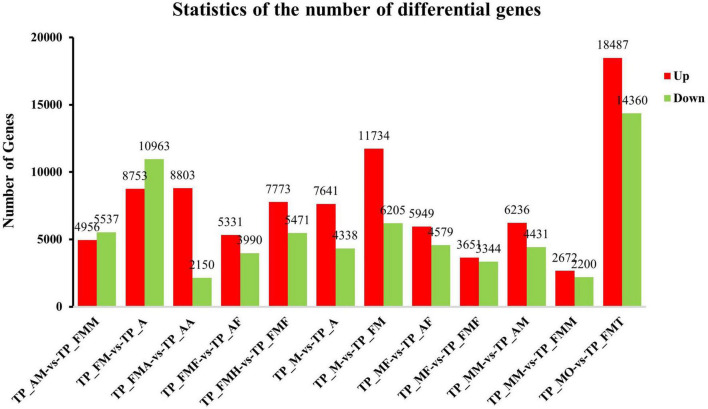
Analysis of differentially expressed genes (DEGs) at different development stage. The X axis represents the difference comparison scheme for each group, and the Y axis represents the corresponding DEG number. Red represents the number of DEGs that are up-regulated, and blue represents the number of DEGs that are down-regulated.

Comparison between different tissue parts and developmental stages showed that the commonly expressed genes were more than 50% of all transcripts obtained. There were 40,826 commonly expressed genes among male and female adults, with 4,310 being female specific genes and 15,512 being male genes (Figure 3A). The nymphs had 37,960 widely expressed genes, among which the least specifically expressed genes were only 1,161 (Figure 3D). Moreover, the nymphs′ antennae specifically express genes (8,005) were more than adult genes (3,291) (Figure 3B). Similarly, testes exhibited more expressed genes (55,753) than the ovaries (38,516). The testes and ovaries had the highest number, 32,847 differential genes (up-regulated 18,487, down-regulated 14,360) (Figures 3C, 4), which accounted for 85.28% of ovary unigenes and 58.96% of the number of testes unigenes. This finding indicated that as much as some of the testes and ovaries-related genes maintain their morphological and physiological functions, the expression patterns of other genes are entirely different. Consistently, comparing the midgut and the fat body of *L. chinensis* at different developmental stages showed that there are fewer specifically expressed genes in females than in nymphs and male adults (midgut 1,867 and Fat body 2,252) (Figures 3E,F) and that the differentially expressed genes in the male and female midguts were the least (4,872). Furthermore, in comparison with nymphs, female adults had 10,493 differentially expressed genes. The differentially expressed genes in adult and larval antennae were 10,953. However, the fat body and nymph comparison indicated that the differentially expressed genes in males were 9,321 and 10,528 in males and females, respectively (Figure 4).

### Differential Analysis of Genes Related to *T. papillosa* Growth and Development

**FIGURE 5:**
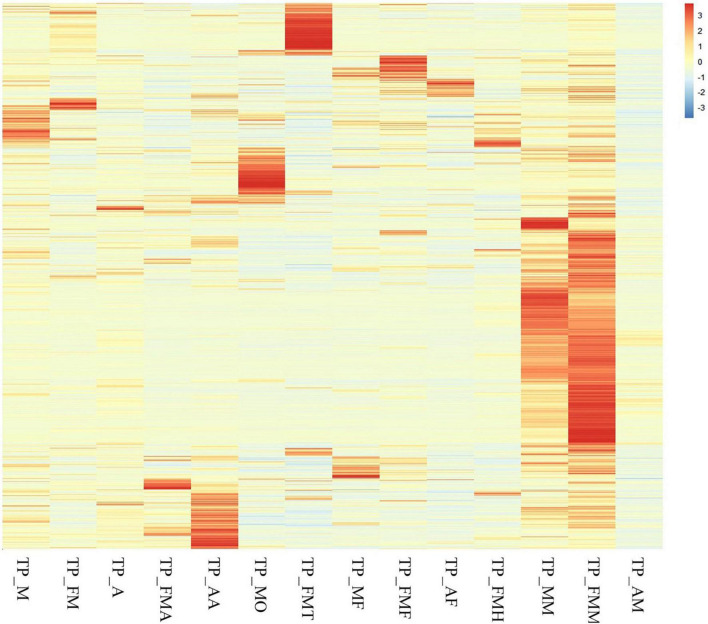
Cluster analysis of all genes among different tissue samples and different developmental stage.

We compared the expression levels of the obtained genes and found that the midgut genes showed a completely different expression pattern from those in the other tissues. In the adult midgut, there were many highly expressed genes (>50%), which was contrary to those observed for nymphs. However, the Nymphs’ antennae, testis, and ovaries had a relatively large number of highly expressed genes (>10%). In the fat body, the highly expressed genes of the three insect states were about 5% but differed between nymphs and males compared to females (Figure 5).

**FIGURE 6:**
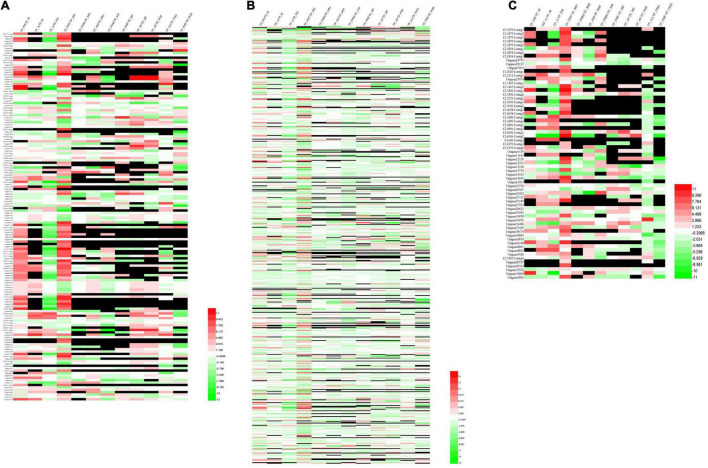
Cluster analysis of differential genes among different tissue samples and different developmental stage. **(A)** Genes related to growth and development. **(B)** Genes of digestion and detoxification enzymes. **(C)** Genes of olfactory.

Upon analysis, 462 growth and development unigenes were obtained, including 58 trypsin, 12 juvenile hormones, 11 ecdysones, 11 chitin, 41 chitinase, 110 cadherin, 7 cuticular proteins, 117 Fatty acdis, and 95 Dynein heavy chains encoding 160 genes (Supplementary Table 4). Additionally, female and male adult worms exhibited noticeable genetic differences between the testes and ovaries. Compared with female adults, nymphs had many silenced genes. Nonetheless, nymph, female and male fat bodies expressed different genes (Figure 6A).

We obtained 1,851 unigenes related to digestion and detoxification. These included 108 Acetyltransferase, 11 Alcohol dehydrogenase, 104 Aminopeptidase, 71 Carboxypeptidase, 95 NADH, 285 Cytochrome, 162 Cytochrome P450, 38 glutathione s-transferase, 57 serine protease, 20 carbonic anhydrases (Carbonic anhydrase), 195 ATPase (ATPase), 56 carboxylases (Carboxylase), 8 acyl-CoA dehydrogenase, 74 cathepsins, 560 protein kinase (protein kinase, 3 aldehyde oxidase, and 4 chymotrypsin, encoding 611 genes (Supplementary Table 5). Moreover, we also observed significant genetic differences between male and female adults. Different genes were also reported in the reproductive system, antennae, and fat body (Figure 6B).

Seventy olfactory unigenes, including 28 odorant-binding proteins (OBPs), 6 chemosensory proteins (CSPs), and 4 pheromone-binding proteins (PBPs), 19 odorant receptors (OR), 11 sensory neuron membrane proteins (SNMPs), and 3 aldehyde oxidase (aldehyde oxidase) unigenes were obtained. Each of the 70 unigenes encoded 10 additional unigenes (Supplementary Table 6). Moreover, cluster analysis of these genes revealed that most olfactory genes are expressed in the testis and ovaries, but with different expression patterns indicating that the olfactory gene expression is regulated during the early developmental stages. Similarly, some olfactory-related genes were also found in the insect midgut (Figure 6C).

**FIGURE 7:**
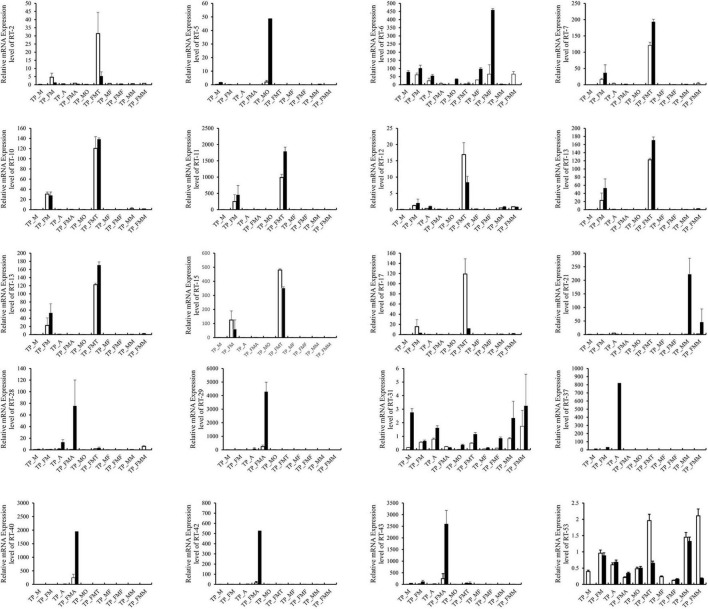
Comparison of qRT-PCR expression level and FPKM expression level of 20 genes in *T. papillosa.* The white column is the qRT-PCR data, and the black column is the RNA-SEQ data.

Based on the differential expression of different tissues in the RNA-SEQ data, we screened 20 genes with significant differences from the digestion and detoxification and olfactory related genes, and used RT-qPCR for gene expression verification (Table 1). The RT-qPCR results were consistent with those obtained through RNA-seq and RNA-SEQ analysis (Figure 7).

**TABLE 1:** qRT-PCR verification of 20 differentially expressed genes.

No.	GeneID	Annotation
RT-2	CL7125.Contig1_Dmry	Dynein heavy chain 3
RT-5	CL5194.Contig2_Dmry	Aminopeptidase N
RT-6	Unigene10305_Dmry	Inter-alpha-trypsin inhibitor heavy chain H3-like isoform X2
RT-7	CL4281.Contig1_Dmry	Cytosol aminopeptidase-like
RT-9	Unigene13392_Dmry	Dynein heavy chain 2
RT-10	CL7507.Contig2_Dmry	Cytosol aminopeptidase-like isoform X1
RT-11	CL204.Contig1_Dmry	Cytochrome P450 6a17
RT-12	Unigene27328_Dmry	Dynein heavy chain 6
RT-13	CL3997.Contig3_Dmry	Cytosol aminopeptidase-like
RT-15	CL328.Contig4_Dmry	Cytochrome P450 4c21-like
RT-17	CL899.Contig4_Dmry	Carnitine O-acetyltransferase-like isoform X1
RT-21	Unigene25423_Dmry	General odorant-binding protein 19d-like
RT-28	Unigene9526_Dmry	Odorant-binding protein 14
RT-29	Unigene14645_Dmry	Odorant-binding protein
RT-31	Unigene32932_Dmry	Chemosensory protein 5
RT-37	Unigene34476_Dmry	General odorant-binding protein 66-like
RT-40	Unigene37429_Dmry	General odorant-binding protein 56d-like
RT-42	CL4896.Contig1_Dmry	General odorant-binding protein 56h
RT-43	CL1836.Contig2_Dmry	General odorant-binding protein 1-like
RT-53	CL5513.Contig1_Dmry	Trypsin-1 isoform X2

## Discussion

During the feeding process, insects take up some secondary plant metabolites together with the nutrients, and these metabolites eventually affect the normal physiological processes of insects (Pan et al., 2016; Yuan et al., 2020). *T. papillosa* is a major pest of longan and lychee trees in Southeast Asia. The infestation results in severe damage to the plants involved. The results show that the midgut of *T. papillosa* has four parts, which is obviously more developed than the midgut system of other insects. At the same time, it also provides the possibility to provide more physical storage. Transcriptome sequencing technology plays a vital role in non-model insect gene discovery and gene function studies. The technology has enabled various species (Bayega et al., 2021; Zhan et al., 2021; Zhao et al., 2021). We performed transcriptome and RNA-SEQ sequencing on the *T. papillosa* genome at different developmental stages and different tissues. This study show that *T. papillosa* and *H. halys* had the highest copies of unigenes. *H. halys* is a polyphagous pest native to East Asia (Sparks et al., 2020; Sun D. D. et al., 2020). Unlike *T. papillosa*, *H. halys* exhibits a wide host range. This may be related to the four stomachs of the *T. papillosa.*

The insects’ detoxification enzymes mainly include cytochrome P450-dependent monooxygenases (P450s), glutathione-S-transferases (GSTs), and carboxylesterases (COE). There are about 150 members detoxification enzymes in insects, and the P450 family members are twice as many as the members of the other two families (Oakeshott et al., 2003; Yuan et al., 2020). Our results also showed that the number and the expression of Cytochrome P450, carboxypeptidase, protein kinase, and other protein family genes were distinct for different physiological periods, especially in the fat body. These genes were closely related to insects’ tolerance and detoxification (Oakeshott et al., 2003; Clarkson et al., 2018). This phenomenon alludes to that the *T. papillosa* has high food utilization efficiency, enhanced degradation capacity of exogenous toxic substances, and increased stress tolerance. Moreover, the reduced food spectrum of the *T. papillosa* also enhances its physiological survival.

Seventy unigenes related to the olfactory-related proteins were obtained, such as OBP and OR, which were lesser than those observed by Wu et al. (2017) in *T. papillosa* antennae analysis (92) (Wu et al., 2017). In other studies, 110 *Apolygus lucorum* antennal transcriptome-related genes (An et al., 2016) and 60 H. *halys* OBPs genes (Paula et al., 2016) were identified. Similarly, the same genes were also highly expressed in different tissues and developmental stages of *T. papillosa*, thus indicating their significance in *T. papillosa* developmental process.

Feeding, reproduction, development, and detoxification processes by *T. papillosa* are physiological responses induced by the complex regulation of multiple genes. Since the insect feeding pattern is regulated by the insect olfactory-related genes, the number of related genes can affect the range of insects’ feeding habits (Li et al., 2013). Concomitantly, stimulation of the environmental factors elicits signals which trigger gene expression, inducing morphological changes of the insects through a series of regulatory processes. The morphology is maintained by a series of genes, which are a result of long-term evolutionary selection. Therefore, most of the genes obtained at different developmental stages through mixed sampling are maintenance-oriented, and only the olfactory, detoxification, and development-related genes of the *T. papillosa* can be utilized at certain stages for preliminary identification and analysis.

The genes with less expression variation are thought to be housekeeping genes associated with basic life activities. Gene expression patterns in the adult midguts were completely different from that of nymphs. Moreover, female fat body genes also displayed distinct expression patterns from those of males and nymphs. These differences may indicate the various physiological activities occurring at different developmental stages. Therefore, the analysis of the physiological functions of the obtained genes provides insights into the development of alternative pest control systems through the molecular screening basis of target genes.

## Conclusion

This study reported the transcriptome data of different developmental stages and different tissues of *T. papillosa*. Through comparative analysis, some genes related to diet, development and host selection were obtained during the entire development process. Through anatomy, it was found that the midgut of the *T. papillosa* consists of four parts (four stomachs). At the same time, the digestive and detoxification enzyme genes in the *T. papillosa* are relatively abundant compared with other types of genes. The findings of these results can provide information for the study of the *T. papillosa*.

## Data Availability Statement

The original contributions presented in the study are publicly available. This data can be found here: PRJNA765492.

## Author Contributions

LC: formal analysis, investigation, and data curation. SH: formal analysis and investigation. JJ: investigation. HL: conceptualization, formal analysis, data curation, and writing—review and editing. LP: conceptualization, data curation, and writing—review and editing. All authors read and approved the final manuscript.

## Conflict of Interest

The authors declare that the research was conducted in the absence of any commercial or financial relationships that could be construed as a potential conflict of interest.

## Publisher’s Note

All claims expressed in this article are solely those of the authors and do not necessarily represent those of their affiliated organizations, or those of the publisher, the editors and the reviewers. Any product that may be evaluated in this article, or claim that may be made by its manufacturer, is not guaranteed or endorsed by the publisher.

## Supplementary Material

Supplementary Figure 1 Distribution map of unigene transcript length and predicted CDS length. **(A)** The abscissa represents the transcript length interval, and the ordinate represents the number of corresponding transcripts. **(B)** The abscissa represents the CDS length interval, and the ordinate represents the number of the corresponding CDS.

Supplementary Figure 2 The number of genes of each transcription factor families.

Supplementary Figure 3 **(A)** Heat map of correlation coefficient between samples. Both X and Y axes represent each sample. The color represents the correlation coefficient (the darker the color, the higher the correlation, and the lighter the color, the lower the correlation). **(B)** Principal component PCA analysis between samples. **(C)** Express volume box plot. The X-axis is the sample name, and the Y-axis is log10 (FPKM+1). The box plot of each region corresponds to five statistics (from top to bottom, the upper limit, upper quartile, median, and lower quartile). Number of digits, lower limit, the upper and lower limits do not consider outliers). **(D)** WGCNA gene module detection. In the figure, a branch of the clustering tree represents a gene, and genes with highly similar expressions are grouped together and belong to the same module (indicated by the same color). The gene correlation between each module is displayed in yellow and red progressive colors. The darker the color (red), the higher the correlation between the two genes.
