# Supplementary material for: Transcriptome Analysis for Identification of Genes Related to Growth and Development, Digestion and Detoxification, Olfaction in the Litchi Stink Bug Tessaratoma papillosa
Source: Front Physiol. 2022 Jan 24;12:774218. doi: 10.3389/fphys.2021.774218 (PMC8818959; doi:10.3389/fphys.2021.774218)
Supplement: Supplementary file 1 [file Data_Sheet_1.docx]

| No. | Sequencing samples | Name |
| --- | --- | --- |
| 1 | Female adult | TP_M |
| 2 | Male adult | TP_FM |
| 8 | Nymph | TP_A |
| 9 | Adult antennae | TP_FMA |
| 10 | Nymph antennae | TP_AA |
| 11 | Ovaries | TP_MO |
| 12 | testis | TP_FMT |
| 13 | Female fat body | TP_MF |
| 14 | Male fat body | TP_FMF |
| 15 | Nymph fat body | TP_AF |
| 17 | Lymph | TP_FMH |
| 18 | Female midgut | TP_MM |
| 22 | Male midgut | TP_FMM |
| 26 | Nymph midgut | TP_AM |

Supplementary Table 1. Samples of different tissues and developmental stages of *T. papillosa*.

Supplementary Table 2. Reads quality statistics after data filtering.

| Sample | Total Raw Reads (M) | Total Clean Reads (M) | Total Clean Bases (Gb) | Clean Reads Q20 (%) | Clean Reads Q30 (%) | Clean Reads Ratio (%) |
| --- | --- | --- | --- | --- | --- | --- |
| TP_A | 21.85 | 21.16 | 1.06 | 98.44 | 91.05 | 96.83 |
| TP_AA | 21.86 | 21.18 | 1.06 | 98.58 | 91.55 | 96.89 |
| TP_AF | 21.85 | 21.17 | 1.06 | 98.54 | 91.32 | 96.89 |
| TP_AM | 21.84 | 21.1 | 1.06 | 98.5 | 91.27 | 96.62 |
| TP_FM | 21.86 | 21.19 | 1.06 | 98.47 | 91.06 | 96.94 |
| TP_FMA | 21.85 | 21.2 | 1.06 | 98.59 | 91.57 | 97.02 |
| TP_FMF | 21.85 | 21.19 | 1.06 | 98.43 | 90.92 | 96.99 |
| TP_FMH | 21.86 | 21.12 | 1.06 | 98.56 | 91.53 | 96.62 |
| TP_FMM | 21.85 | 21.16 | 1.06 | 98.56 | 91.57 | 96.86 |
| TP_FMT | 24 | 23.06 | 1.15 | 98.52 | 91.4 | 96.1 |
| TP_M | 21.85 | 21.01 | 1.05 | 98.58 | 91.73 | 96.17 |
| TP_MF | 21.85 | 21.16 | 1.06 | 98.35 | 90.61 | 96.86 |
| TP_MM | 21.85 | 21.17 | 1.06 | 98.38 | 90.84 | 96.88 |
| TP_MO | 21.83 | 21.04 | 1.05 | 98.6 | 91.67 | 96.41 |

Supplementary Table 3. Reference gene result statistics.

| Sample | Total Clean Reads (M) | Total Mapping (%) | Uniquely Mapping (%) |
| --- | --- | --- | --- |
| TP_A | 21.16 | 92.28 | 54.79 |
| TP_AA | 21.18 | 88.92 | 56.91 |
| TP_AF | 21.17 | 94.03 | 52.38 |
| TP_AM | 21.1 | 94.07 | 49.43 |
| TP_FM | 21.19 | 93.78 | 54.33 |
| TP_FMA | 21.2 | 93.89 | 25.57 |
| TP_FMF | 21.19 | 96.05 | 48.4 |
| TP_FMH | 21.12 | 94.05 | 53.98 |
| TP_FMM | 21.16 | 93.79 | 52.6 |
| TP_FMT | 23.06 | 94.48 | 36.2 |
| TP_M | 21.01 | 94.13 | 56.31 |
| TP_MF | 21.16 | 95.53 | 53.95 |
| TP_MM | 21.17 | 94.56 | 53.81 |
| TP_MO | 21.04 | 95.57 | 47.18 |
