# Supplementary figures and images for: Transcriptome Analysis for Identification of Genes Related to Growth and Development, Digestion and Detoxification, Olfaction in the Litchi Stink Bug Tessaratoma papillosa
Source: Front Physiol. 2022 Jan 24;12:774218. doi: 10.3389/fphys.2021.774218 (PMC8818959; doi:10.3389/fphys.2021.774218)

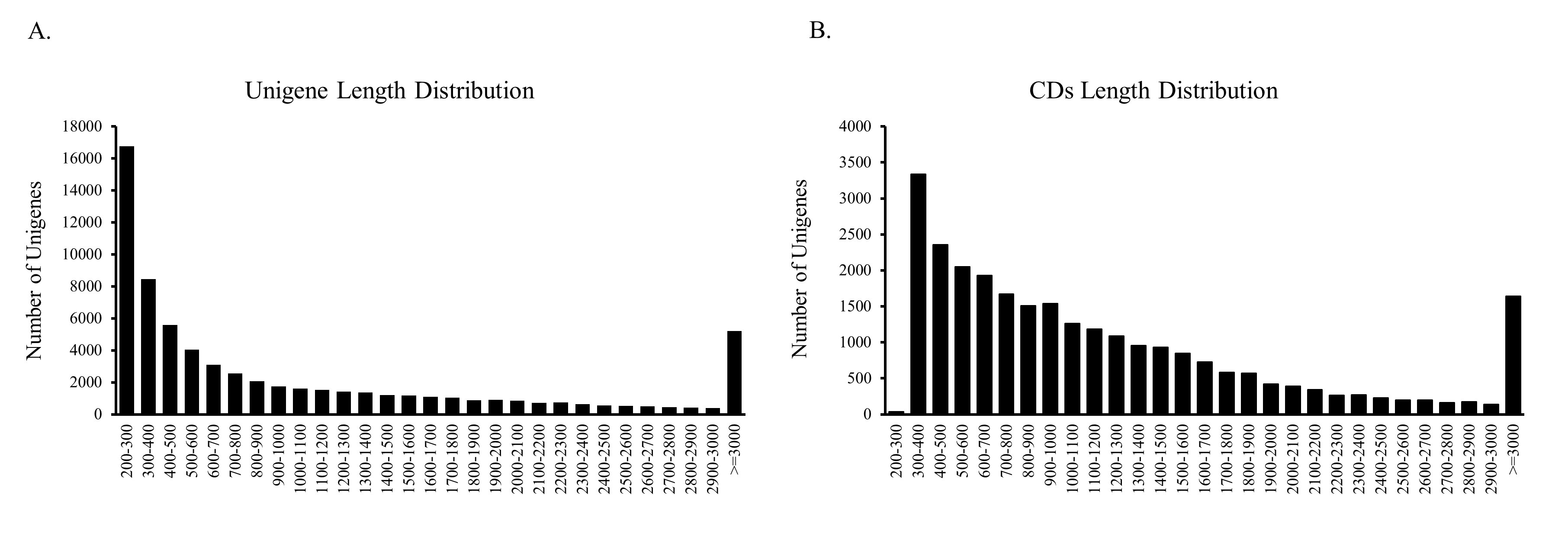

Supplement: Supplementary Figure 1 — Distribution map of unigene transcript length and predicted CDS length. (A) The abscissa represents the transcript length interval, and the ordinate represents the number of corresponding transcripts. (B) The abscissa represents the CDS length interval, and the ordinate represents the number of the corresponding CDS. [file Image_1.JPEG]

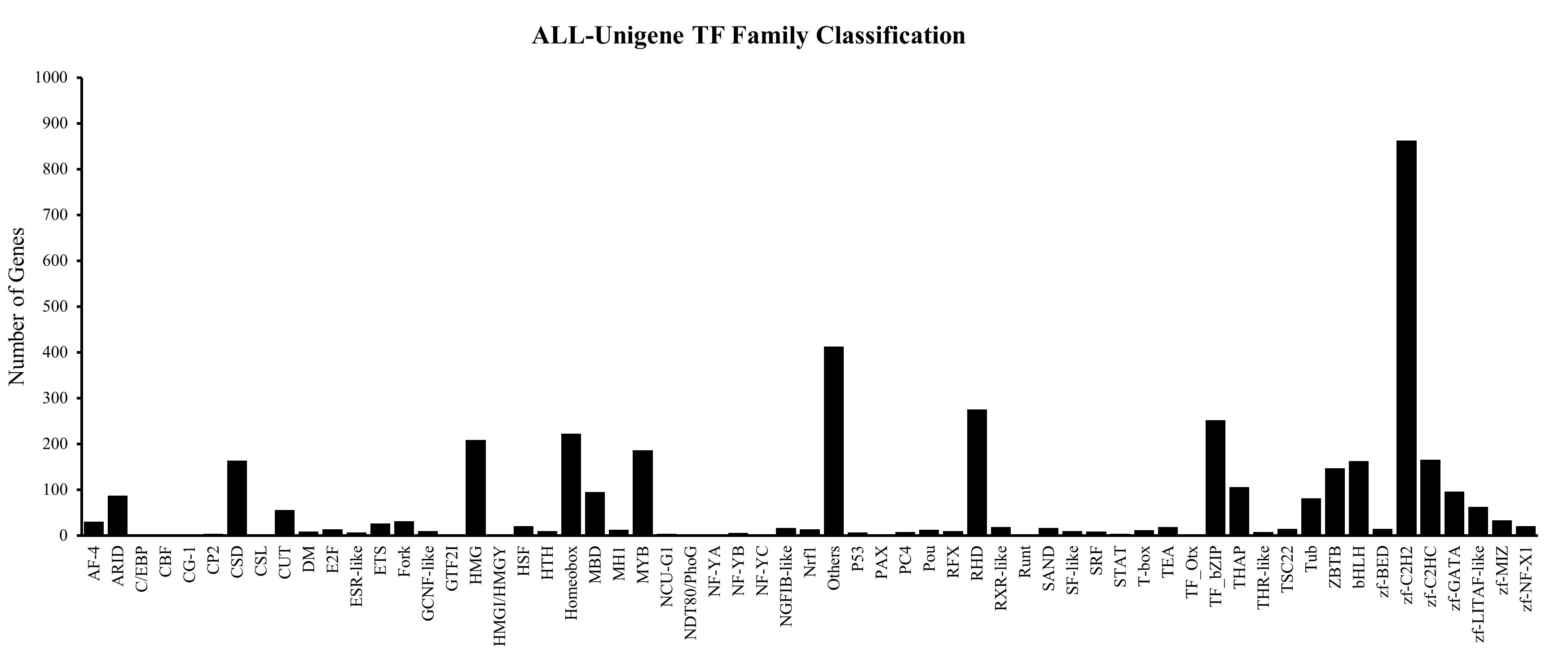

Supplement: Supplementary Figure 2 — The number of genes of each transcription factor families. [file Image_2.JPEG]

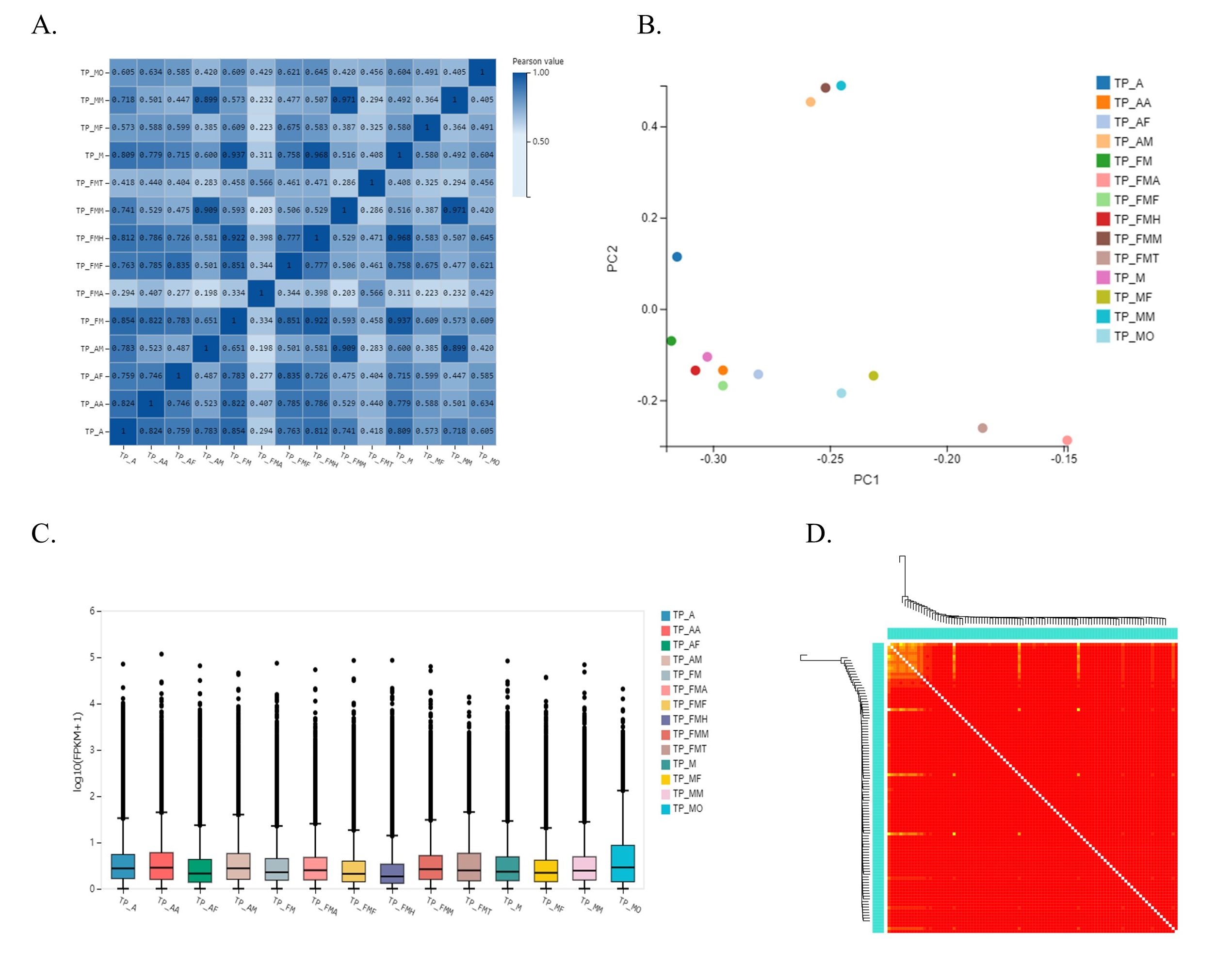

Supplement: Supplementary Figure 3 — (A) Heat map of correlation coefficient between samples. Both X and Y axes represent each sample. The color represents the correlation coefficient (the darker the color, the higher the correlation, and the lighter the color, the lower the correlation). (B) Principal component PCA analysis between samples. (C) Express volume box plot. The X-axis is the sample name, and the Y-axis is log10 (FPKM+1). The box plot of each region corresponds to five statistics (from top to bottom, the upper limit, upper quartile, median, and lower quartile). Number of digits, lower limit, the upper and lower limits do not consider outliers). (D) WGCNA gene module detection. In the figure, a branch of the clustering tree represents a gene, and genes with highly similar expressions are grouped together and belong to the same module (indicated by the same color). The gene correlation between each module is displayed in yellow and red progressive colors. The darker the color (red), the higher the correlation between the two genes. [file Image_3.JPEG]
